# Nutritional status indices on the prognosis of patients with relapsed and refractory multiple myeloma treated with CAR-T cell immunotherapy

**DOI:** 10.3389/fnut.2025.1654407

**Published:** 2025-10-01

**Authors:** Peng Xu, Yang Liu, Yawen Wang, Qiwen Feng, Huanxin Zhang, Hai Cheng, Kunming Qi, Kailin Xu, Zhenyu Li

**Affiliations:** ^1^Blood Disease Institute, Xuzhou Medical University, Xuzhou, Jiangsu, China; ^2^Department of Hematology, The Affiliated Hospital of Xuzhou Medical University, Xuzhou, Jiangsu, China; ^3^Jiangsu Key Laboratory of Bone Marrow Stem Cells, Xuzhou, Jiangsu, China

**Keywords:** controlling nutritional status, prognostic nutritional index, chimeric antigen receptor T cell, multiple myeloma, prognosis

## Abstract

**Background aims:**

Chimeric antigen receptor (CAR) T-cell therapy shows remarkable efficacy against relapsed/refractory multiple myeloma (R/R MM). Nutritional status, assessed by objective indices like the Controlling Nutritional Status (CONUT) and Prognostic Nutritional Index (PNI), influences MM prognosis. However, their predictive value for outcomes following CAR-T therapy in R/R MM remains unclear.

**Methods:**

We conducted a retrospective analysis of 181 R/R MM patients receiving CAR-T therapy. Patients were stratified by optimal CONUT (cutoff: 6.5) and PNI (cutoff: 42.75) scores determined via ROC analysis. Associations between CONUT/PNI and treatment outcomes were investigated.

**Results:**

Patients with low CONUT or high PNI exhibited significantly improved progression-free survival (PFS) and overall survival (OS) compared to their counterparts (high CONUT or low PNI). While objective response rates (ORR) were high overall (approximately 90–95%), they did not differ significantly between CONUT or PNI subgroups. Importantly, low CONUT or high PNI was associated with faster hematopoietic recovery (red blood cells, hemoglobin, platelets, neutrophils, CD4+, CD8 + T-cells), lower incidence of prolonged hematologic toxicity (PHT), and higher peak CAR transgene levels. No significant differences were observed in cytokine release syndrome (CRS) or immune effector cell-associated neurotoxicity syndrome (ICANS) severity between subgroups. Multivariate analysis confirmed high CONUT as an independent risk factor for inferior PFS and OS, while low PNI was an independent risk factor for inferior PFS.

**Conclusion:**

This study establishes pretreatment CONUT and PNI as significant prognostic predictors for R/R MM patients undergoing CAR-T therapy. Patients with low CONUT or high PNI experience superior long-term survival outcomes, potentially linked to enhanced hematopoietic recovery and CAR-T cell expansion. These findings underscore the importance of nutritional assessment in prognostication and may guide future strategies to optimize CAR-T outcomes in R/R MM.

## Introduction

Multiple myeloma (MM), the second most common hematologic malignancy, is a clonal plasma cell disorder characterized by monoclonal protein in the blood or urine and associated organ damage, including hypercalcemia, renal insufficiency, anemia, or bone lesions (1, 2). Over recent decades, the integration of proteasome inhibitors (PIs), immunomodulatory drugs (IMiDs), and monoclonal antibodies (mAbs) into combination regimens—as initial therapy with hematopoietic stem cell transplantation (HCT) for eligible patients, and as continuous therapy for transplant-ineligible patients—has significantly improved overall survival and progression-free survival in multiple myeloma (3–5). However, relapsed/refractory (R/R) MM remains a challenge, as a significant proportion of patients fail to benefit. Chimeric antigen receptor (CAR) T-cell therapy is an emerging immunotherapy for the treatment of R/R MM and has achieved surprising efficacy (6–8). Nevertheless, the prognosis for patients with R/R MM following CAR-T therapy varies significantly. As nutritional status is a crucial factor influencing outcomes in cancer patients (9), conducting effective nutritional assessments in this population is essential.

The controlling nutritional status (CONUT), an indicator of immune-nutritional status calculated from serum albumin concentration, peripheral blood lymphocyte count, and total cholesterol, serves as a screening tool for early detection of malnutrition and is associated with the prognosis of esophageal and lung cancers (10, 11). The prognostic nutritional index (PNI), composed of serum albumin levels and absolute lymphocyte count, reflects a patient’s immune and nutritional status. Initially used to assess preoperative nutritional status, surgical risk, and postoperative complications in surgical patients, it has now been applied in oncology for diseases such as breast cancer, lung cancer, and lymphoma (12–14). Both CONUT and PNI are objective and simple indices for evaluating nutritional status. Notably, our research team previously investigated the impact of Body Mass Index (BMI) on the prognosis of R/R MM patients receiving CAR-T cell therapy (15). The study revealed that a higher BMI was associated with longer progression-free survival (PFS). However, BMI primarily reflects the quantitative relationship between weight and height. In contrast, composite nutritional-immunological indices such as CONUT and PNI incorporate biomarkers like serum albumin and lymphocyte counts. These indices assess patient status from an integrative metabolic and immune function perspective, thus providing additional prognostic dimensions not captured by BMI alone.

Recent studies have confirmed that high CONUT scores and low PNI scores are significantly associated with poorer overall survival and could predict the prognosis of MM patients (16, 17). A study by Özkan et al. (18) showed that the CONUT can serve as a valuable predictive tool for early complications after MM transplantation, thereby guiding targeted interventions and improving patient management. However, studies on the prognostic effect of CONUT and PNI on adoptive cell therapy (ACT) are limited, and the effect of CONUT and PNI on the efficacy and prognosis of patients with R/R MM administered CAR-T cell therapy has not been confirmed. In this study, a retrospective analysis was performed to investigate the effect of CONUT and PNI on CAR-T cell therapy outcomes in patients with R/R MM.

## Patients and methods

### Study population

This retrospective study included 181 consecutive patients with R/R MM treated at the Affiliated Hospital of Xuzhou Medical University between June 2018 and February 2023. Among them, 89 patients were overlapping with the cohort of our previous study [June 2017–February 2022 (15)], and 92 new patients (March 2022–February 2023) were added to expand the sample size. The baseline characteristics of the new patients (e.g., median age, R-ISS stage, type of CAR-T cell therapy, and prior therapy lines) were consistent with the overlapping population (Supplementary Table S2). Additionally, to address potential bias introduced by the 89 patients overlapping with our prior BMI study, we performed a sensitivity analysis in the non-overlapping cohort (*n* = 92). Results showed that in this non-overlapping cohort, the correlations between CONUT/PNI and key outcomes (PFS, OS, incidence of PHT, and peak CAR-T levels) remained statistically significant, confirming the robustness of our findings (Supplementary Table S3) All patients received BCMA-targeted CAR-T cell therapy administered as: (1) single-target BCMA CAR-T cells, (2) combined sequential infusion of BCMA and CD19 CAR-T cells (BCMA + CD19), or (3) bispecific tandem BCMA/CD19 CAR-T cells (BC19). The study was approved by the hospital’s Ethics Committee and conducted in accordance with the Declaration of Helsinki, with all participants providing written informed consent. The trial was registered on Chictr.org.cn (ChiCTR2000033567, ChiCTR-OIC-17011272, and ChiCTR1900026219). Detailed inclusion and exclusion criteria align with previous publications (19, 20). All patients underwent lymphodepletion with fludarabine (30 mg/m^2^/d, days −5 to −3) and cyclophosphamide (750 mg/m^2^/d, day −5).

### Assessments of nutritional status

In this study, we used two nutritional indices to assess the nutritional status of the population: CONUT and PNI. Their respective calculation methodologies are detailed in Table 1.

**Table 1:** Details of the nutritional indices utilized in the study.

Score	Abbreviation	Calculation formula	Reference
Prognostic nutritional index	PNI	Serum albumin (g/L) + (5 × lymphocytes (× 10^9^/L))	(40)
Controlling nutritional status	CONUT	Serum albumin score + total lymphocyte count score + total cholesterol score. For serum albumin levels > 35, between 30 and 34, between 25 and 29 and <25 g/L, 0, 2, 4, and 6 points were assigned, respectively. For serum total cholesterol levels > 180, between 140 and 179, between 100 and 139 and < 100 mg/dL, 0, 1, 2, and 3 points were assigned, respectively. For serum total lymphocyte count > 1.6, between 1.2 and 1.6, between 0.8 and 1.1 and <0.8 × 109/L, 0, 1, 2, and 3 points were assigned, respectively.	(41)

### Data collection

Patient demographic and disease characteristics collected at enrollment included age, gender, myeloma type, prior treatment history, tumor burden, cytogenetic abnormalities, and extramedullary disease status. Post-CAR-T infusion laboratory parameters—including blood cell counts, lymphocyte subsets, and peak CAR transgene expansion—were compared between nutritional subgroups stratified by CONUT and PNI scores.

### Definition and follow-up

Prolonged hematologic toxicity (PHT) was defined as Grades 3–4 cytopenia persisting beyond day 28. Cytokine release syndrome (CRS) and immune effector cell-associated neurotoxicity syndrome (ICANS) severity was graded retrospectively according to American Society for Transplantation and Cellular Therapy (ASTCT) consensus criteria (21). The response of patients with R/RMM to CAR-T cell infusion was evaluated based on the International Myeloma Working Group Revised Uniform Response (2014) criteria (22). Overall survival (OS) spanned from CAR-T infusion to death from any cause. Progression-free survival (PFS) was measured from CAR-T infusion to disease progression or death. Overall response rate (ORR) represented the proportion of patients achieving ≥ partial response (PR). All patients were followed through June 30, 2023, with data sourced from inpatient/outpatient records and telephone follow-ups.

### Statistical methods

Statistical analyses were performed using GraphPad Prism v8.0. Continuous variables are expressed as median (range) and compared using Mann–Whitney U or independent *t*-tests based on distribution normality. Categorical variables are presented as *n* (%) and analyzed by *χ*^2^ or Fisher’s exact tests. Covariate-adjusted comparisons employed analysis of covariance (ANCOVA). Survival distributions were estimated via Kaplan–Meier curves with between-group differences assessed by log-rank test. Univariate and multivariate Cox proportional hazards models identified factors associated with OS and PFS. All statistical tests were two-sided with significance defined as *p* < 0.05.

## Results

### Determination of the cut-off value and clinical characteristics

A total of 181 patients with R/R MM were enrolled in this study. According to receiver operating characteristic (ROC) curve analysis, the areas under the curves (AUC) for CONUT and PNI were 0.6699 and 0.6551, with *p*-values of 0.0003 and 0.0009, respectively (Supplementary Figure S1). Although the AUC values were modest (<0.70), they remained statistically significant and aligned with prior studies in hematologic malignancies where AUCs of 0.60–0.70 are clinically accepted for prognostic indices in heterogeneous cohorts (23, 24). The cut-off value for CONUT was 6.5 (95% CI 0.581–0.759), with a sensitivity of 68% and specificity of 71.43%; the cut-off value for PNI was 42.75 (95% CI 0.590–0.740), with a sensitivity of 62.4% and specificity of 64.29%. These thresholds were selected to maximize Youden’s index and are consistent with established cutoffs in MM studies: Özkan et al. used CONUT≥5 for early post-HCT complications (18), while Kamiya et al. (25) defined high CONUT as >4 for OS prediction in MM, supporting the clinical relevance of our threshold. Patients were divided into low CONUT group (≤6.5, 101 cases, 55.8%) and high CONUT group (>6.5, 80 cases, 44.2%), and into low PNI group (≤42.75, 83 cases, 45.86%) and high PNI group (>42.75, 98 cases, 54.14%).

In this study cohort, the median patient age was 58 years (range: 34–75). Males comprised 97 cases (53.6%). According to the R-ISS staging system, 112 patients (61.88%) were classified as stage III. Regarding M-protein subtype, the IgG type accounted for 108 cases (59.67%). In terms of treatment, 57 patients received BCMA CAR-T cell therapy, 68 patients received combined BCMA CAR-T cell and CD19 CAR-T cell therapy (BCMA + CD19), and 56 patients received tandem BCMA/CD19 CAR-T cell therapy (BC19). The baseline characteristics and parameters of the patients are detailed in Table 2.

**Table 2:** Demographics and baseline disease characteristics before CAR-T cell therapy.

Characteristic	Total (*n* = 181)	CONUT			PNI		
		≤6.5 (*n* = 101)	>6.5 (*n* = 80)	*P*	≤42.75 (*n* = 83)	>42.75 (*n* = 98)	*P*
Age, median (range)	58 (34–75)	57 (34–72)	58 (42–75)	0.613	58 (45–75)	58 (34–69)	0.718
Male, no. (%)	97 (53.59)	56 (55.45)	41 (51.25)	0.653	39 (46.99)	58 (59.18)	0.135
R-ISS stage III, no. (%)	112 (61.88)	68 (67.33)	44 (55)	0.094	53 (63.86)	59 (60.2)	0.647
Type of myeloma, no. (%)							
IgG	108 (59.67)	56 (55.45)	52 (65)	0.223	49 (59.04)	59 (60.2)	0.88
Non-IgG							
IgA	28 (15.47)	16 (15.84)	12 (15)		15 (18.07)	13 (13.27)	
IgD	16 (8.84)	11 (10.89)	5 (6.25)		7 (8.43)	9 (9.18)	
Light chain	22 (13.81)	13 (12.87)	9 (11.25)		9 (10.84)	13 (13.27)	
Non-secretory	7 (3.88)	5 (4.95)	2 (2.5)		3 (3.61)	4 (4.08)	
Type of CAR-T cell therapy, no. (%)				0.186			0.287
BCMA	57 (31.49)	26 (25.74)	31 (38.75)		22 (26.51)	35 (35.71)	
BCMA+ CD19	68 (37.57)	41 (40.59)	27 (33.75)		31 (37.35)	37 (37.76)	
Tandem BC19	56 (3.39)	34 (33.66)	22 (27.5)		30 (36.14)	26 (26.53)	
High tumor burden, no. (%)^a^	42 (23.2)	19 (18.81)	23 (28.75)	0.156	26 (31.33)	16 (16.33)	**0.022**
High-risk cytogenetic features, no. (%)^b^	44 (24.31)	21 (20.79)	23 (28.75)	0.227	15 (18.07)	29 (29.59)	0.083
Extramedullary lesions, no. (%)^c^	61 (33.7)	38 (37.62)	23 (28.75)	0.268	27 (32.53)	34 (34.69)	0.875
Previous therapy lines, median (range)	4 (1–12)	4 (1–12)	4 (2–10)	0.53	4 (2–12)	4 (1–12)	0.76
Previous HCT, no. (%)	63 (34.81)	28 (27.72)	35 (43.75)	**0.028**	38 (45.78)	25 (25.51)	**0.005**

BCMA, B-cell maturation antigen; BCMA+ CD19, combined anti-BCMA and anti-CD19 CAR-T cells; Tandem BC19, tandem BCMA/CD19 CAR-T cells; HCT, stem cell transplantation; R-ISS, revised international staging system.

^a^High tumor burden: defined as ≥50% clonal plasma cells or bone marrow plasma cells.

^b^High-risk cytogenetics was reported by investigators based on fluorescence in situ hybridization. A high-risk cytogenetic profile was defined by the presence of the following abnormalities: del (17p), *t* (4; 14), or t (14; 16).

^c^Extramedullary diseases included tissue masses in extraosseous locations and bone-related plasmacytomas.

Bold type highlights statistically significant results (defined as *P* < 0.05 using the Wald test).

Correlation analysis with patients’ clinical characteristics showed that the low PNI group had a higher tumor burden compared to the high PNI group, and this difference was statistically significant (*p* = 0.022). We also found that whether patients had undergone prior HCT showed statistically significant differences in both the CONUT (*p* = 0.028) and PNI (*p* = 0.005) subgroups. Other common clinical characteristics showed no statistically significant differences in the CONUT and PNI subgroups, as detailed in Table 2.

### Correlation with ORR

**Figure 1:**
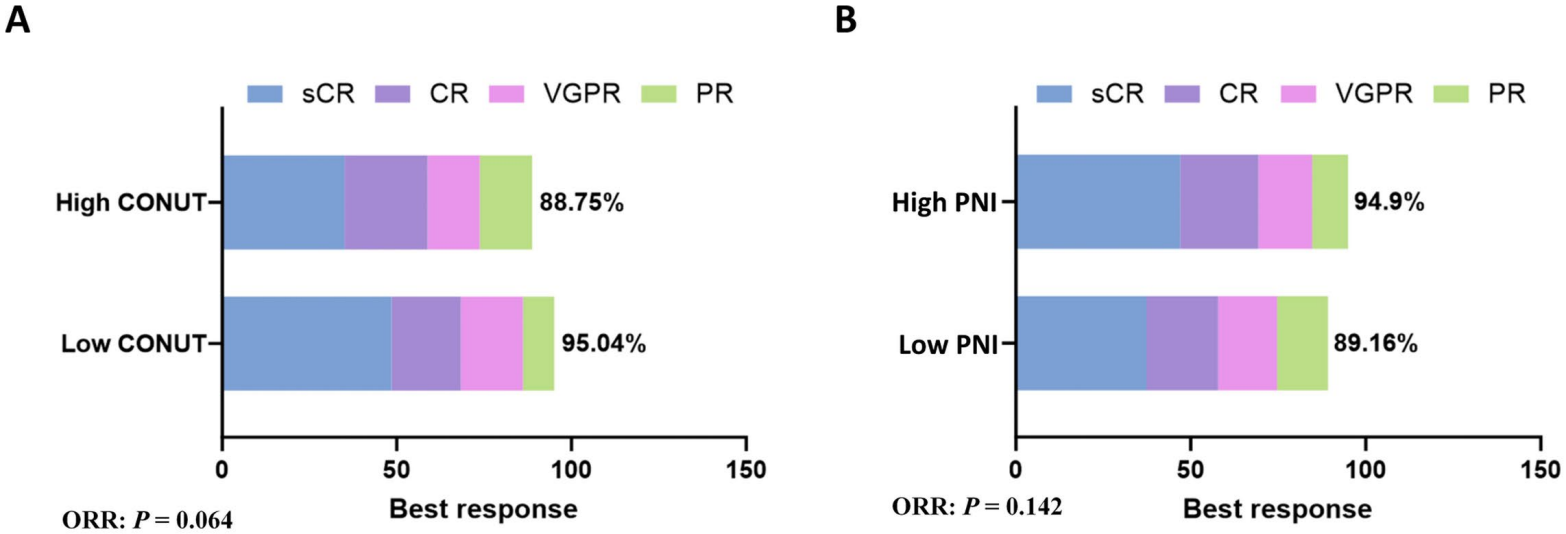
Subgroup analysis of ORR. **(A)** The rates of best response in CONUT subgroups. **(B)** The rates of best response in PNI subgroups.

In the low CONUT group, 96 patients achieved a response, yielding an ORR of 95.04%. This included 49 patients (48.51%) with stringent complete response (sCR), 20 patients (19.8%) with complete response (CR), 18 patients (17.82%) with very good partial response (VGPR), and 9 patients (8.91%) with partial response (PR). In the high CONUT group, 71 patients achieved a response, yielding an ORR of 88.75%. This included 28 patients (35%) with sCR, 19 patients (23.75%) with CR, 12 patients (15%) with VGPR, and 12 patients (15%) with PR. There was no significant difference in ORR between the low CONUT and high CONUT groups (*p* = 0.064) (Figure 1A).

In the low PNI group, 74 patients achieved a response, yielding an ORR of 89.16%. This included 31 patients (37.35%) with sCR, 17 patients (20.48%) with CR, 14 patients (16.87%) with VGPR, and 12 patients (14.46%) with PR. In the high PNI group, 93 patients achieved a response, yielding an ORR of 94.9%. This included 46 patients (46.94%) with sCR, 22 patients (22.45%) with CR, 15 patients (15.31%) with VGPR, and 10 patients (10.2%) with PR. There was no significant difference in ORR between the low PNI and high PNI groups (*p* = 0.142) (Figure 1B).

### Correlation with OS or PFS

Follow-up was conducted until June 30, 2023. The median follow-up time after CAR-T cell therapy was 19.2 months (95% confidence interval [CI]: 18.91–26.15). In the low CONUT group, the median OS was not reached, and the median PFS was 17.9 months (95% CI: 12.31–28.49). In the high CONUT group, the median OS was 46.20 months (95% CI: not reached [NR]) and the median PFS was 13.4 months (95% CI: 6.32–15.21) (Table 3).

**Table 3:** Univariate and multivariate analysis of PFS and OS.

Parameter	Progression-free survival (PFS)				Overall survival (OS)			
	Univariate analysis		Multivariate analysis		Univariate analysis		Multivariate analysis	
	HR (95% CI)	*P*	HR (95% CI)	*P*	HR (95% CI)	*P*	HR (95% CI)	*P*
Age, >60 years	0.824 (0.625–1.371)	0.517			0.808 (0.452–1.053)	0.674		
Gender, male	1.031 (0.728–2.415)	0.741			1.098 (0.167–3.982)	0.533		
R-ISS stage III	1.924 (1.734–3.417)	**0.028**	1.261 (0.528–1.397)	0.089	1.836 (0.917–2.438)	0.061		
Type of myeloma, IgG	1.251 (0.528–2.185)	0.692			1.059 (0.592–2.071)	0.544		
Type of CAR-T cell therapy		0.315				0.613		
BCMA (reference)								
BCMA+ CD19	1.136 (0.713–2.359)	0.279			1.212 (0.357–2.195)	0.433		
Tandem BC19	1.341 (0.751–2.752)	0.253			1.635 (0.577–3.822)	0.528		
High tumor burden	1.042 (1.003–1.762)	**0.019**	1.102 (1.039–2.491)	**0.033**	1.366 (1.128–1.956)	**0.022**	1.285 (1.033–2.397)	**0.027**
High-risk cytogenetic features	1.453 (1.137–2.195)	**0.042**	1.094 (0.831–2.752)	0.172	1.285 (0.544–2.972) 0.135			
Extramedullary lesions	1.147 (1.092–1.891)	**0.023**	1.067 (1.003–2.966)	**0.041**	1.877 (1.262–2.496)	**0.039**	1.655 (0.876–3.422)	0.078
Prior lines of therapy, ≥4	1.652 (0.316–2.137)	0.215			1.498 (0.574–1.933) 0.376			
Previous HCT	1.473 (0.902–2.863)	0.078			1.289 (0.873–2.185) 0.192			
CONUT score, >6.5	1.734 (1.319–3.463)	**0.008**	1.359 (1.038–3.417)	**0.003**	1.433 (1.097–2.188)	**0.017**	1.355 (1.088–2.977)	**0.023**
PNI score, ≤42.75	1.451 (1.093–2.196)	**0.012**	1.192 (1.087–2.981)	**0.029**	1.349 (1.034–2.977)	**0.031**	1.297 (0.958–2.733)	0.055

Bold type highlights statistically significant results (defined as *P* < 0.05 using the Wald test).

**Figure 2:**
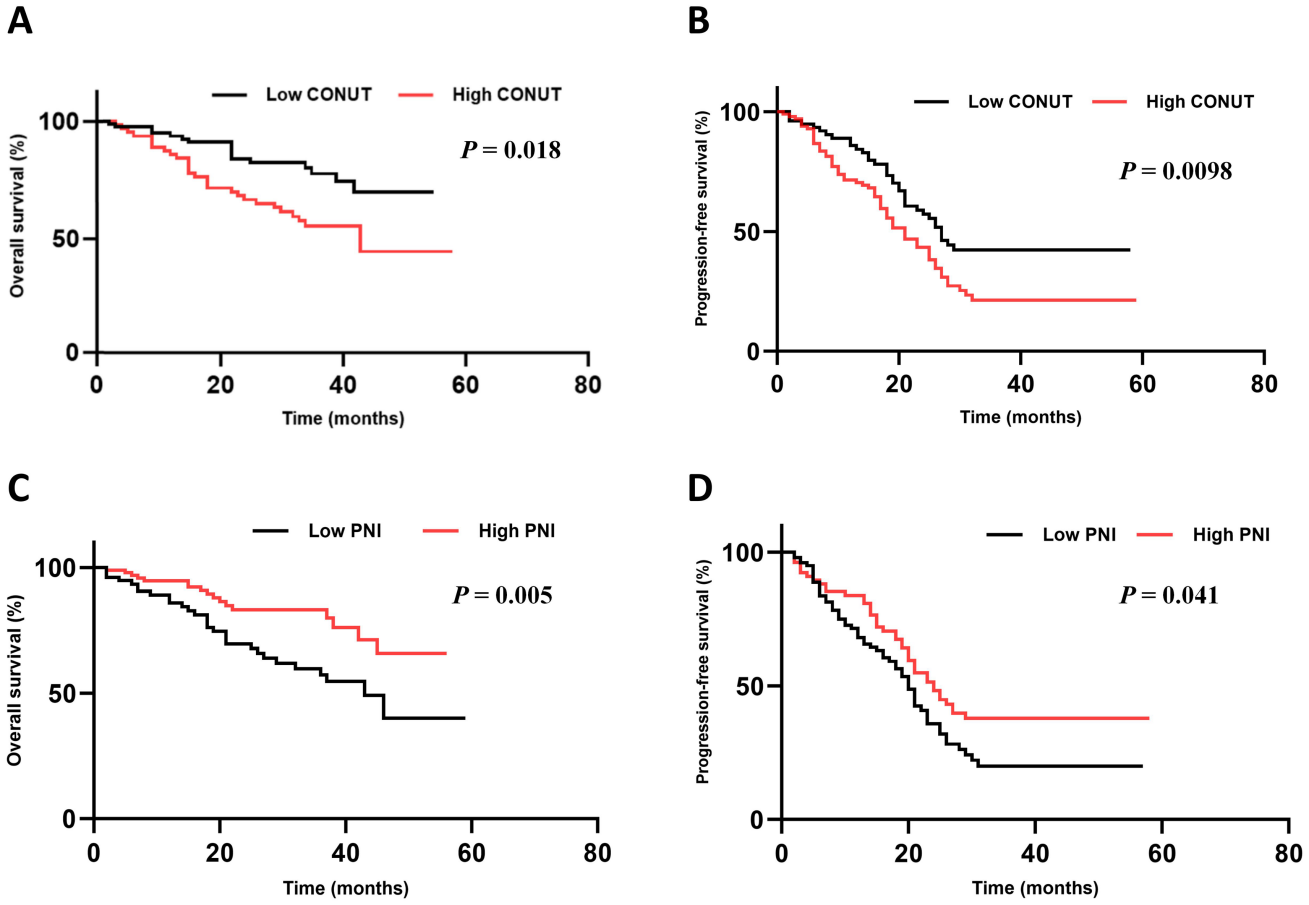
Subgroup analysis of OS and PFS. **(A,B)** The OS and PFS in CONUT subgroups. **(C,D)** The OS and PFS in PNI subgroups.

Both OS (*p* = 0.018) and PFS (*p* = 0.0098) were significantly superior in the low CONUT group compared to the high CONUT group (Figures 2A,B).

In the low PNI group, the median OS was 44.30 months (95% CI: NR) and the median PFS was 14.7 months (95% CI: 11.58–26.12). In the high PNI group, the median OS was not reached, and the median PFS was 11.6 months (95% CI: 7.18–14.62). Both OS (*p* = 0.005) and PFS (*p* = 0.041) were significantly superior in the high PNI group compared to the low PNI group (Figures 2C,D).

### Factors associated with PFS and OS

Univariate analysis showed that R-ISS stage (*p* = 0.028), high tumor burden (*p* = 0.019), High-risk cytogenetic features (*p* = 0.042), extramedullary lesions (*p* = 0.023), CONUT (*p* = 0.008), and PNI (*p* = 0.012) were influencing factors for PFS in patients with R/RMM. Variables with statistical significance in the univariate regression analysis were included in the multivariate analysis. The results showed that high tumor burden (*p* = 0.033), extramedullary lesions (*p* = 0.041), CONUT (*p* = 0.003), and PNI (*p* = 0.029) were independent risk factors for PFS in R/R MM patients receiving CAR-T cell therapy (Table 2).

Univariate analysis showed that high tumor burden (*p* = 0.022), extramedullary lesions (*p* = 0.039), CONUT (*p* = 0.017), and PNI (*p* = 0.031) were influencing factors for OS in R/R MM patients. Variables with statistical significance in the univariate regression analysis were included in the multivariate analysis. The results showed that high tumor burden (*p* = 0.027) and CONUT (*p* = 0.023) were independent risk factors for OS in R/R MM patients receiving CAR-T cell therapy (Table 2).

### Correlation with CRS and ICANS

The CRS and ICANS are the most common inflammation-related complications after CAR-T cell infusion. In this study, there was no significant difference in CRS grade (*p* = 0.61) or ICANS grade (*p* = 0.631) between the CONUT subgroups. Similarly, there was no significant difference in CRS grade (*p* = 0.437) or ICANS grade (*p* > 0.999) between the PNI subgroups (Supplementary Table S1).

### Correlation with cytopenia

**Figure 3:**
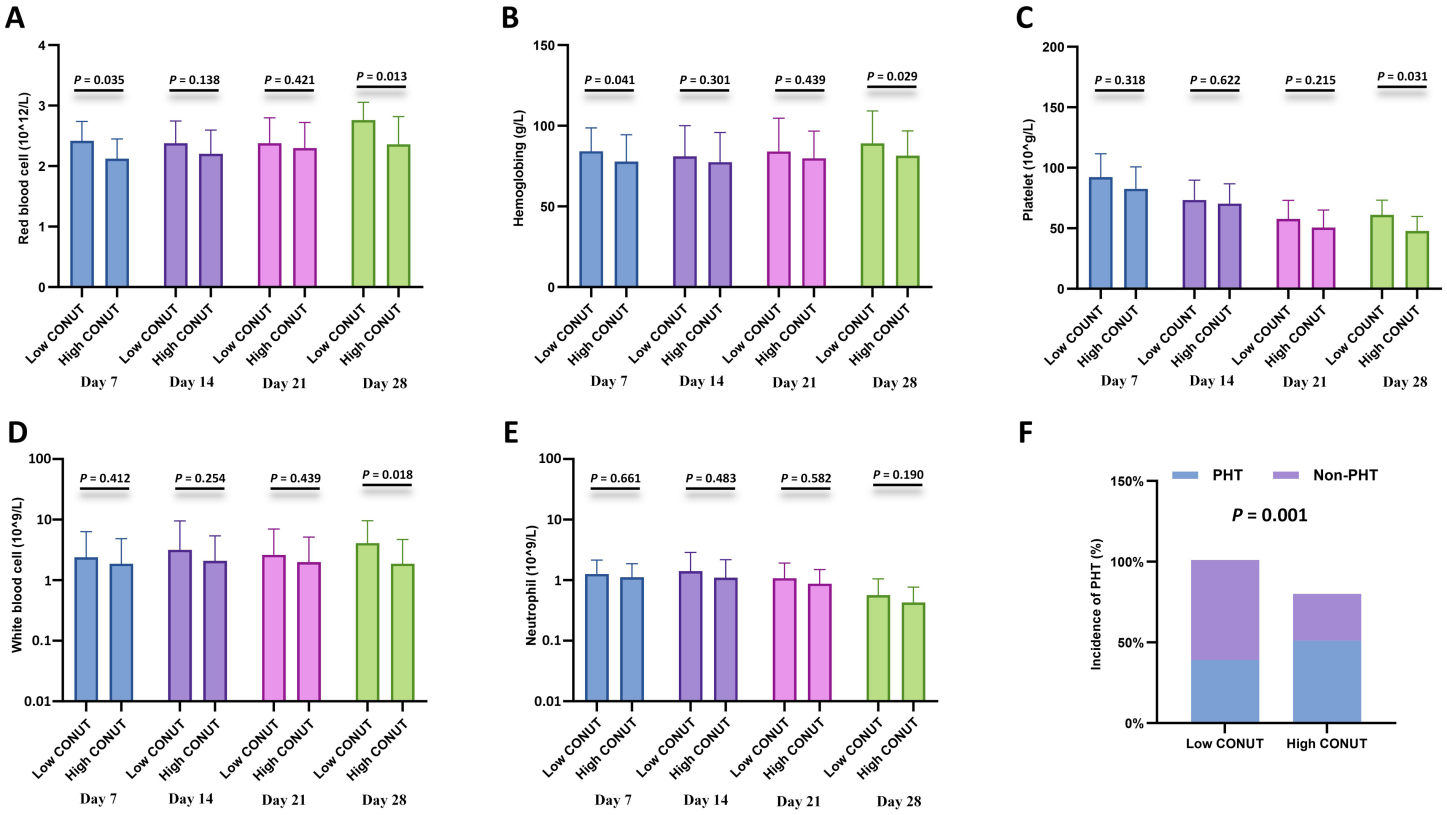
Hematologic parameters and prolonged hematologic toxicity (PHT) stratified by CONUT. **(A–E)** Comparisons of red blood cell (RBC) count (×10^12^/L), hemoglobin (Hb) level (g/L), platelet (PLT) count (×10^9^/L), white blood cell (WBC) count (×10^9^/L), and neutrophil count (×10^9^/L) between the low CONUT group (≤6.5) and high CONUT group (>6.5) at 7, 14, 21, and 28 days after CAR-T cell infusion. **(F)** Incidence of PHT (defined as Grade 3–4 cytopenia persisting beyond day 28) in the low vs. high CONUT subgroups.

**Figure 4:**
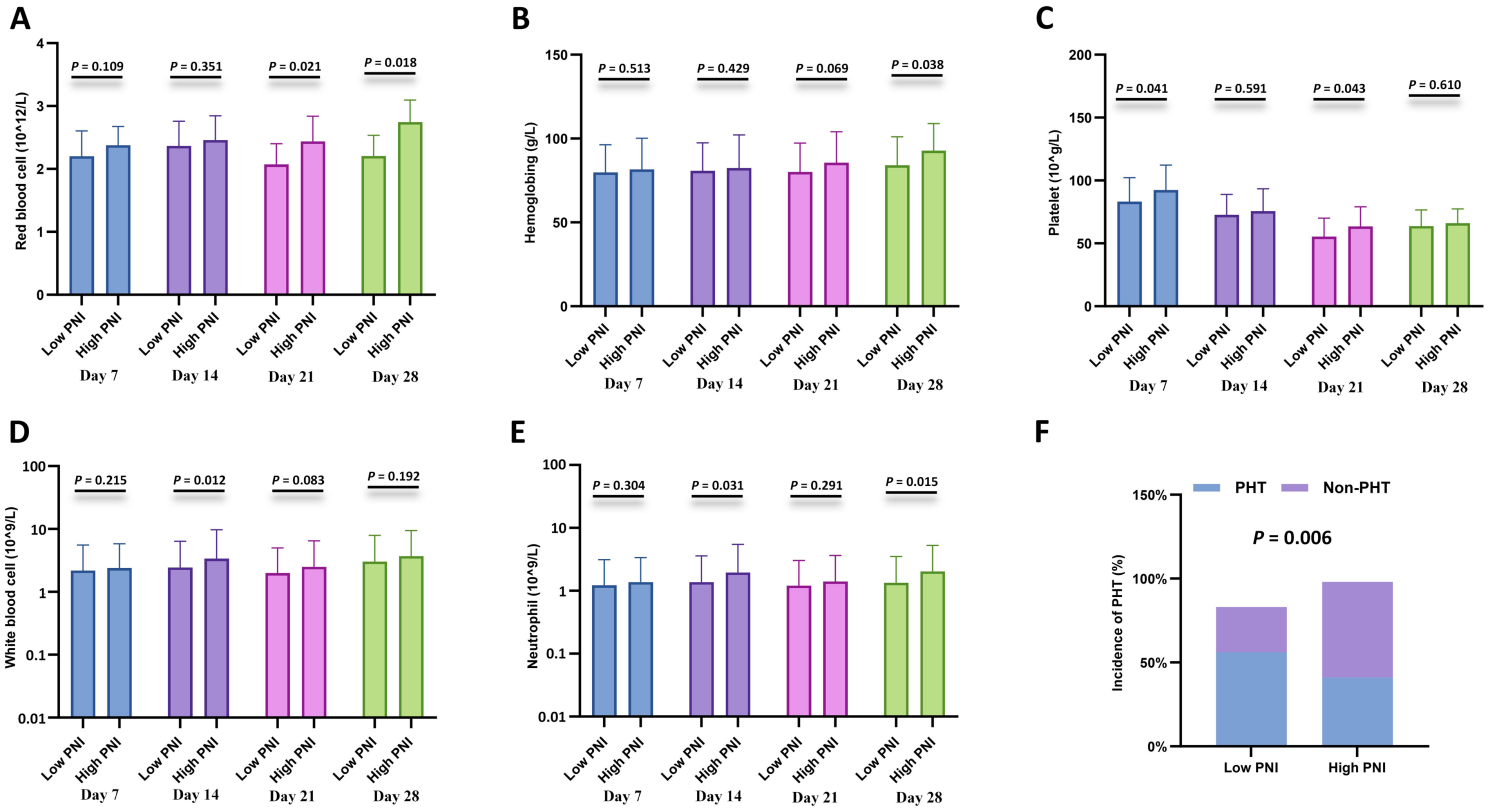
Hematologic parameters and prolonged hematologic toxicity (PHT) stratified by PNI. **(A–E)** Comparisons of red blood cell (RBC) count (×10^12^/L), hemoglobin (Hb) level (g/L), platelet (PLT) count (×10^9^/L), white blood cell (WBC) count (×10^9^/L), and neutrophil count (×10^9^/L) between the low CONUT group (≤6.5) and high CONUT group (>6.5) at 7, 14, 21, and 28 days after CAR-T cell infusion. **(F)** Incidence of PHT (defined as Grade 3–4 cytopenia persisting beyond day 28) in the low vs. high CONUT subgroups.

Furthermore, cytopenia is another common complication after CAR-T cell infusion. Peripheral blood cell counts at different time points after CAR-T cell infusion (days 7, 14, 21, and 28) were compared between the CONUT subgroups and between the PNI subgroups, respectively. In the CONUT subgroups, the low CONUT group had significantly higher values than the high CONUT group for: red blood cell (RBC) count on day 7 and day 28, hemoglobin (Hb) level on day 7 and day 28, platelet (PLT) count on day 28, white blood cell (WBC) count on day 28 (*p* < 0.05 for all) (Figures 3A–E). In the PNI subgroups: The high PNI group had significantly higher values than the low PNI group for: RBC count on day 21 and day 28, Hb level on day 28, PLT count on day 7 and day 21, WBC count on day 14, Neutrophil count on day 14 and day 28 (*p* < 0.05 for all) (Figures 4A–E). There were no significant differences in blood cell counts at the other specified time points between either the CONUT or PNI subgroups (Figures 3, 4).

Additionally, correlation analysis with PHT showed that the incidence of PHT was significantly lower in the low CONUT group compared to the high CONUT group (*p* = 0.001) (Figure 3F), and significantly lower in the high PNI group compared to the low PNI group (*p* = 0.006) (Figure 4F).

### Correlation with lymphocyte subsets

**Figure 5:**
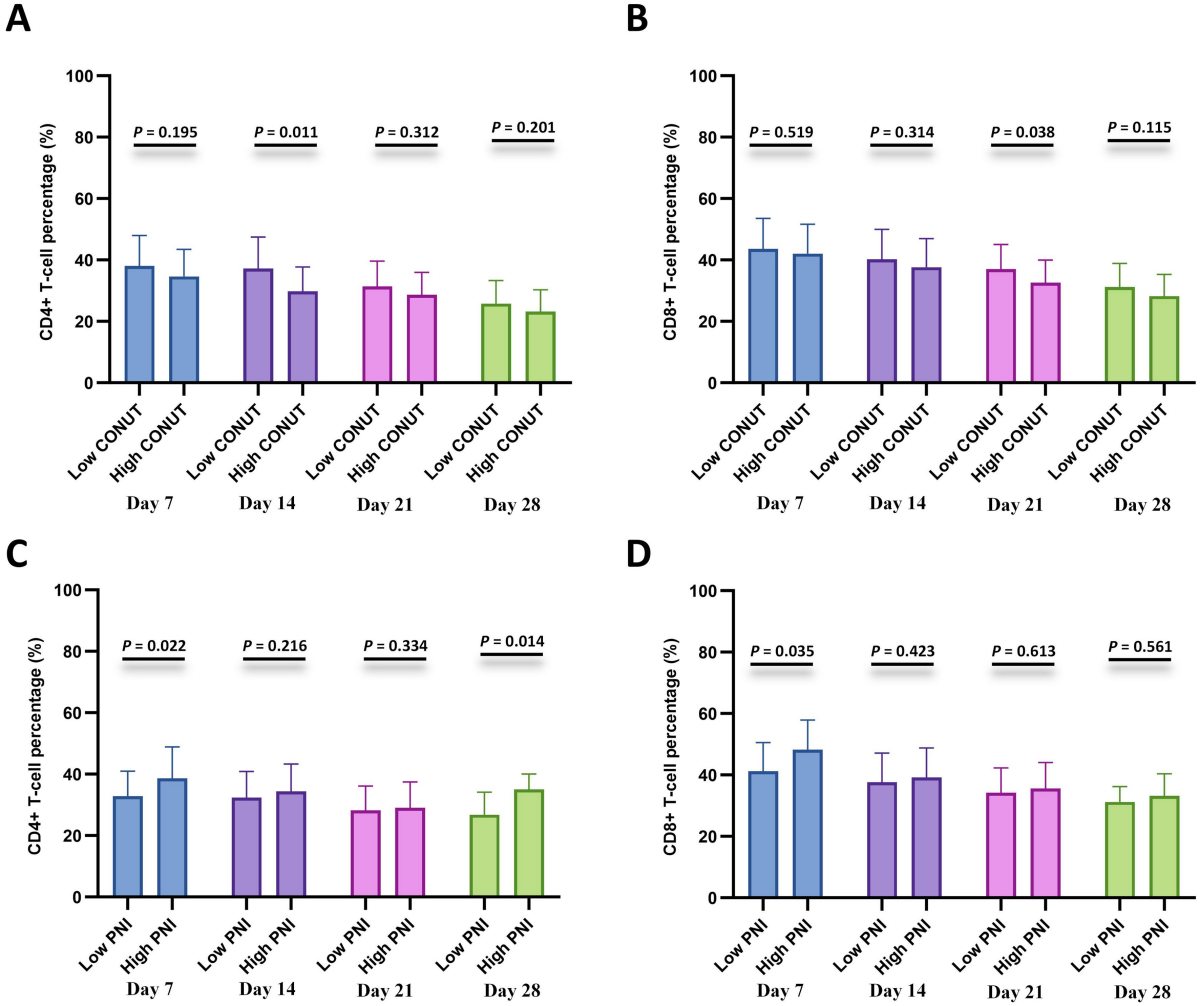
Lymphocyte subset recovery stratified by CONUT and PNI. **(A,B)** Comparisons of CD4 + T-cell percentage (%) and CD8 + T-cell percentage (%) between the low CONUT group (≤6.5) and high CONUT group (>6.5) at 7, 14, 21, and 28 days after CAR-T cell infusion. **(C,D)** Comparisons of CD4^+^ T-cell percentage (%) and CD8^+^ T-cell percentage (%) between the low PNI group (≤42.75) and high PNI group (>42.75) at 7, 14, 21, and 28 days after CAR-T cell infusion.

To further investigate the relationship with cellular immunity, we analyzed CD4 + and CD8 + T-cell percentages at specified time points after CAR-T cell infusion (days 7, 14, 21, and 28) between the CONUT subgroups and between the PNI subgroups, respectively. The results showed that the low CONUT group exhibited significantly higher CD4 + T-cell percentages on day 14 and CD8 + T-cell percentages on day 21 compared to the high CONUT group (*p* < 0.05) (Figures 5A,B). The high PNI group showed significantly higher CD4 + T-cell percentages on day 7 and day 28 and CD8 + T-cell percentages on day 7 compared to the low PNI group (*p* < 0.05) (Figures 5C,D).

### Correlation with the peak levels of CAR transgene

**Figure 6:**
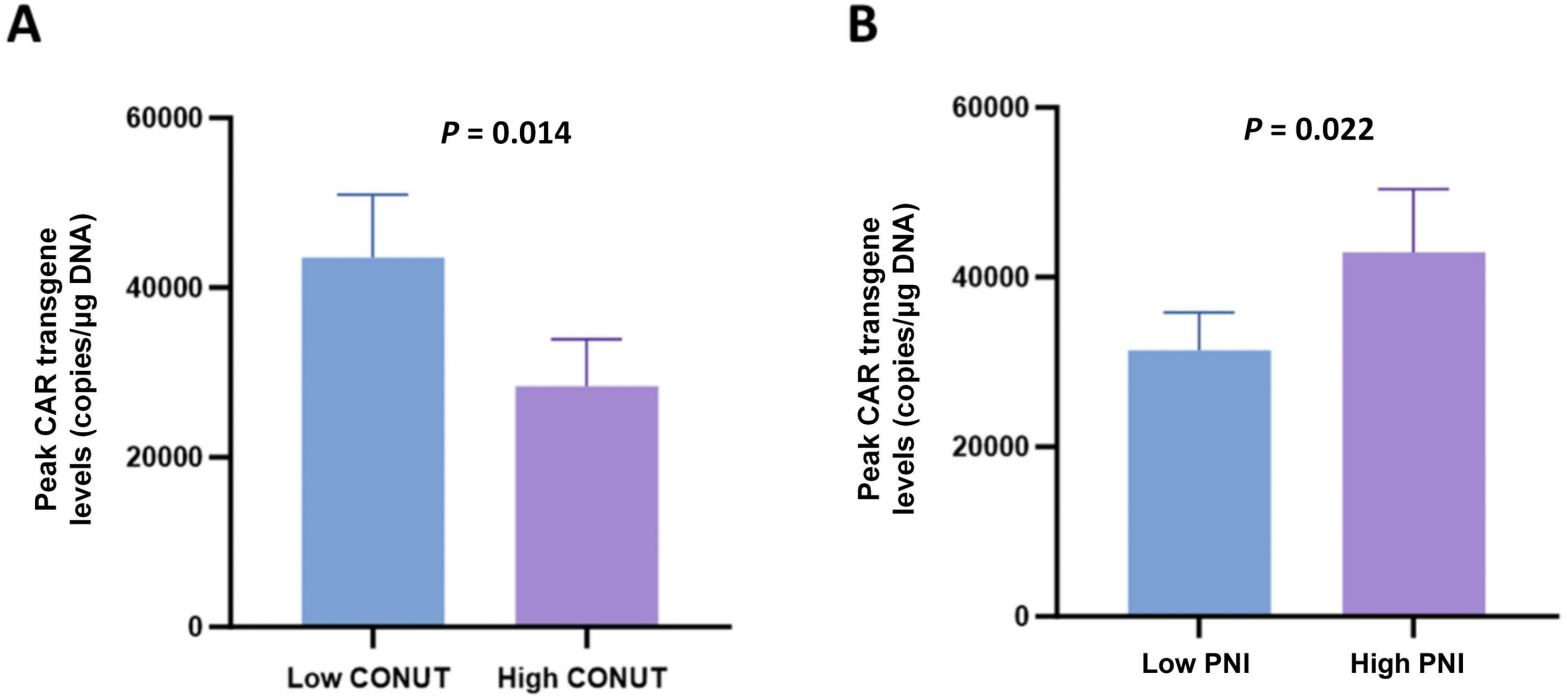
Peak levels of CAR transgene in CONUT and PNI subgroups. **(A)** Comparison of peak CAR transgene levels (copies/μg DNA) between the low CONUT group (≤6.5) and high CONUT group (>6.5). **(B)** Comparison of peak CAR transgene levels (copies/μg DNA) between the low PNI group (≤42.75) and high PNI group (>42.75).

Analysis of the peak levels of CAR transgene among CONUT and PNI subgroups revealed that patients in the low CONUT group and high PNI group had significantly higher peak levels of CAR transgene compared to the high CONUT group and low PNI group, respectively, with statistically significant differences (Figures 6A,B).

## Discussion

Over the past decade, CAR-T cell therapy has made significant advances in the treatment of hematologic malignancies, showing immense promise particularly for R/R MM. Successive studies have revealed that nutritional status is closely correlated with the occurrence and prognosis of cancer patients. Both the CONUT and PNI score are convenient to calculate and clinically practical, effectively reflecting the body’s nutritional status. They have garnered widespread attention for predicting objective indicators like survival in certain cancer patients. However, research on the impact of CONUT and PNI on the prognosis and efficacy of CAR-T therapy for R/R MM is currently limited. This study retrospectively analyzed the clinical characteristics of 181 R/R MM patients who received CAR-T cell therapy. Firstly, ROC curves were used to determine the optimal cut-off values for CONUT and PNI before CAR-T treatment. The relationship between patient clinical characteristics and CONUT/PNI was then analyzed. Patients in the high PNI and low CONUT group demonstrated longer PFS and OS compared to those in the high CONUT and low PNI group. Furthermore, both PNI and CONUT were identified as independent prognostic factors for OS in R/R MM patients. Previous studies indicate that CONUT and PNI serve as simple, reliable prognostic indicators for hematologic malignancies such as diffuse large B-cell lymphoma and plasma cell neoplasm, with low PNI or high CONUT scores predicting poor prognosis (25–27). These findings are consistent with our results. Several potential mechanisms have been proposed for this association, including improved tolerance to chemotherapy, reduced risk of secondary infections, and differences in chemotherapy metabolism (28–30).

Notably, the progression from monoclonal gammopathy of undetermined significance (MGUS) to MM is strongly influenced by both tumor-intrinsic factors and the bone marrow microenvironment (BME). The BME not only supports malignant plasma cell survival and proliferation but also contributes to immune evasion and drug resistance through complex cellular (e.g., stromal cells, immunosuppressive myeloid populations) and molecules (e.g., cytokine gradients, metabolic signaling) interactions (31). In this context, nutritional status (assessed by CONUT/PNI) may serve as a surrogate marker of overall immune competence and systemic resilience, which are partly shaped by the host-microenvironment interplay. Our findings that low CONUT and high PNI correlate with better CAR-T outcomes align with this notion, as these indices likely reflect reduced systemic and microenvironmental dysregulation, enabling more effective antitumor responses.

We also assessed the correlation between CONUT/PNI and cytopenia after CAR-T cell infusion. The results showed that within 30 days of post-infusion, the high PNI and low CONUT group had higher blood cell counts compared to the low PNI and high CONUT group. Furthermore, the incidence of PHT was lower in the high PNI and low CONUT group than in the high CONUT and low PNI group. Previous studies have indicated that better nutritional status facilitates the recovery of peripheral blood cells after chemotherapy (18, 32). A study on CAR-T cell therapy for R/R lymphoma patients demonstrated that the PFS of the early non-severe cytopenia group was significantly higher than that of the severe cytopenia group (33). Additionally, a prior study from our center on CAR-T cell therapy for R/R MM patients revealed that patients with PHT had worse PFS and OS compared to those without PHT (34). Therefore, the higher peripheral blood cell counts observed in the high PNI and low CONUT group after CAR-T cell therapy may be one of the contributing factors to their survival benefit.

Moreover, we also found that compared to the high CONUT and low PNI group, the high PNI and low CONUT group exhibited higher peak levels of CAR transgene. We further investigated the association between CONUT/PIN and toxicity complications related to immunotherapy. The results showed no significant differences in CRS or ICANS grades among the PNI and CONUT subgroups. Hence, part of the reason for the better prognosis in R/R MM patients who received CAR-T cell therapy with better nutritional status may be their ability to achieve higher peak levels of CAR transgene without inducing more severe adverse reactions.

Infection is an important factor that reduces the quality of life and affects the prognosis of MM patients. Patients with R/R MM experience cellular immunodeficiency after CAR-T cell infusion, which is a well-established risk factor for both common and opportunistic infections (35). Furthermore, previous research has shown that lower proportions of CD4 + T cells and CD8 + T cells in MM patients may be closely associated with disease progression and poor prognosis (36). In this study, we found that the low CONUT groups and high PNI group exhibited faster recovery of both CD4 + T-cells and CD8 + T-cells. Some studies have confirmed that T cells secrete large amounts of cytokines, thereby enhancing CAR-T cell infiltration and the immune-mediated clearance of tumors (37–39). Therefore, more rapid immune cell reconstitution may represent another reason for the improved long-term survival observed in patients with better nutritional status after CAR-T cell therapy.

Emerging evidence highlights that BME components can impair T-cell activation and function, limiting the efficacy of immunotherapies such as CAR-T cells (31). In R/R MM, clonal evolution and sustained therapeutic pressure further disrupt immune surveillance. Nutritional-immune indices such as CONUT and PNI may indirectly capture the e degree of systemic and microenvironmental dysregulation: for instance, low PNI (reflecting hypoalbuminemia and lymphopenia) or high CONUT (indicating impaired protein metabolism and immune depletion) could signal a BME enriched for immunosuppressive cells or cytokines that hinder CAR-T infiltration and activity. This is supported by our observation that high PNI and low CONUT groups exhibit higher peak CAR transgene levels and accelerated recovery of CD4 + and CD8 + T cells. These findings implicate nutritional fitness in enhanced CAR-T expansion and immune reconstitution, potentially overcoming BME-mediated immunosuppression. Therefore, in clinical practice, implementing stratified nutritional interventions guided by CONUT and PNI is recommended. For patients with high CONUT or low PNI, preoperative interventions such as albumin supplementation and nutritional-immune formulations (e.g., those containing arginine and ω-3 fatty acids) could be considered to enhance nutritional-immune status. However, the optimal timing and specific protocols for these interventions require further validation.

Compared with our previous findings on BMI (15), the current study further revealed that nutritional status (assessed by CONUT/PNI) is associated with CAR-T cell expansion and hematologic recovery: patients with low CONUT or high PNI had higher peak CAR transgene levels (*p* = 0.002 and *p* = 0.005, respectively) and lower incidence of prolonged hematologic toxicity (*p* = 0.001 and *p* = 0.006, respectively), which were not observed in the BMI-based analysis. These results suggest that nutritional-immune indices may better reflect the body’s tolerance to CAR-T therapy than a single physical index like BMI.

This study has several limitations. First, as a retrospective single-center study, it is susceptible to selection and information biases, and the lack of an external validation cohort affects the robustness of the conclusions. Second, the study only evaluated baseline CONUT and PNI without incorporating analysis of their dynamic changes, and failed to thoroughly explore the impacts of comorbidities, nutritional interventions, and heterogeneity of different CAR-T products. Finally, although the cut-off values derived from ROC curves achieved statistical significance within this cohort, their clinical utility is constrained by the modest discriminatory power (AUC < 0.70). Future multi-center prospective studies with larger independent cohorts, dynamic nutritional monitoring, and evaluation of nutritional intervention effects are needed to validate and extend the findings of this study.

## Conclusion

In summary, our findings demonstrate that pretreatment CONUT and PNI are significant prognostic predictors for R/R MM patients receiving CAR-T cell immunotherapy, with low CONUT or high PNI associated with better long-term prognosis. These indices likely reflect systemic immune integrity and BME status, providing insight into patients’ ability to mount effective antitumor responses. Integrating CONUT and PNI into clinical risk stratification may optimize therapeutic decision-making and patient management.

## Data Availability

The raw data supporting the conclusions of this article will be made available by the authors, without undue reservation.
