# Supplementary material for: Nutritional status indices on the prognosis of patients with relapsed and refractory multiple myeloma treated with CAR-T cell immunotherapy
Source: Front Nutr. 2025 Oct 1;12:1654407. doi: 10.3389/fnut.2025.1654407 (PMC12520877; doi:10.3389/fnut.2025.1654407)
Supplement: Supplementary file 1 [file Table_1.docx]

**Figure S1. ROC curve analysis of the CONUT (A) and PNI (B) score for predicting OS in 181 patients with R/R MM.**

**
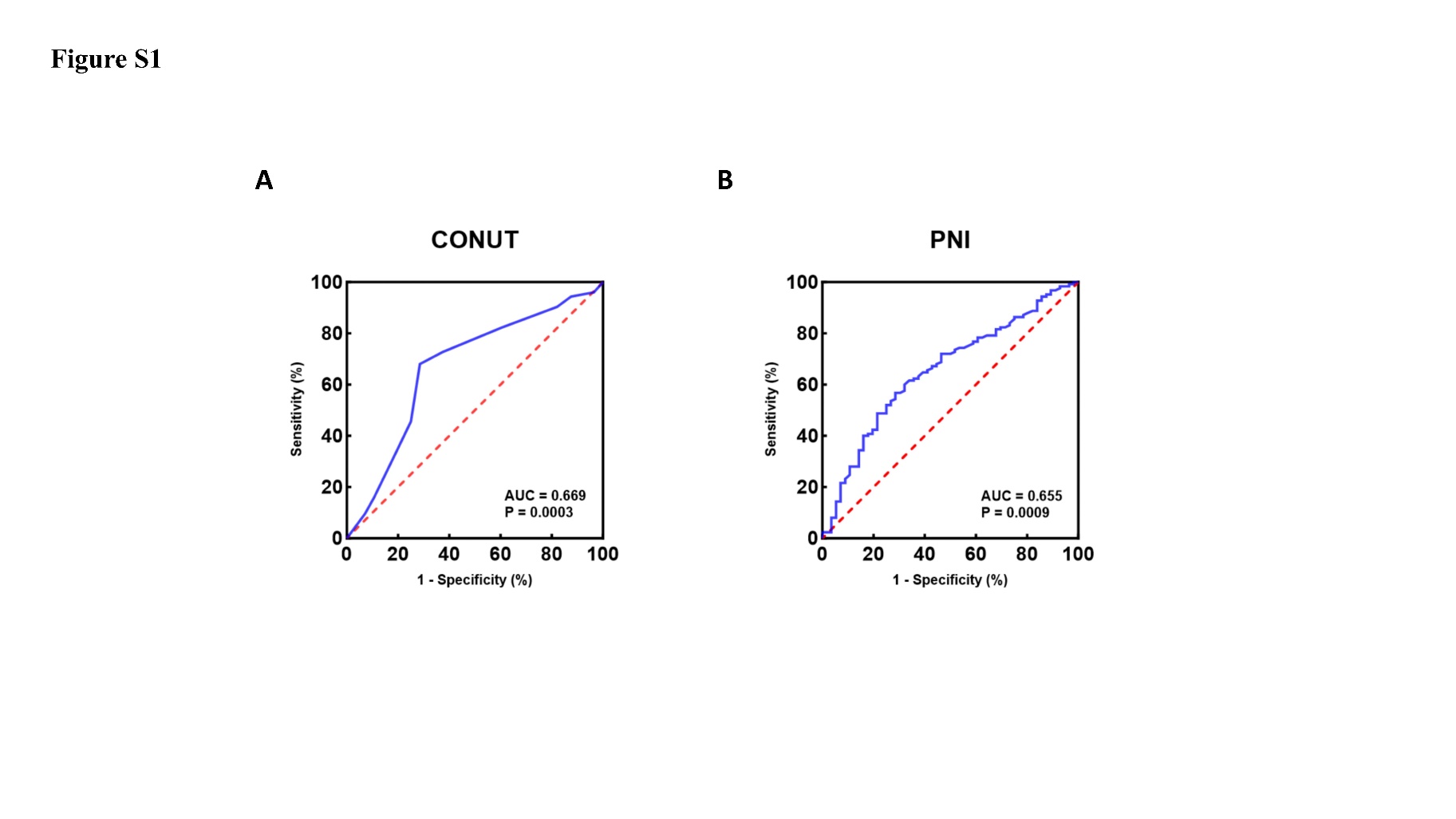
**

**Table S1** CRS and ICANS grades between the CONUT and PNI subgroups.

|  |  | CONUT | | | PNI | | |
| --- | --- | --- | --- | --- | --- | --- | --- |
|  | Total（n = 181） | ≤ 6.5（n = 101） | ＞ 6.5（n = 80） | *P* | ≤ 42.75（n = 83） | ＞ 42.75（n = 98） | *P* |
| CRS grades |  |  |  | 0.61 |  |  | 0.437 |
| 1-2 | 165 | 91 | 74 |  | 74 | 91 |  |
| 3-5 | 16 | 10 | 6 |  | 9 | 7 |  |
| ICANS grades |  |  |  | 0.631 |  |  | ＞0.999 |
| 0 | 173 | 96 | 77 |  | 80 | 93 |  |
| 1-2 | 5 | 4 | 1 |  | 2 | 3 |  |
| 3-5 | 3 | 2 | 1 |  | 1 | 2 |  |

**Table S2.** Baseline characteristics of overlapping and newly added patients.

| Characteristic | Overlapping patients (n = 89) | Newly added patients (n = 92) | *P* |  |
| --- | --- | --- | --- | --- |
| Age, median (range) | 56 (29 - 72) | 58 (34 - 75) | 0.613 |  |
| Male, no. (%) | 52 (58.4) | 45 (48.9) | 0.167 |  |
| R-ISS stage III, no. (%) | 58 (65.2) | 54 (58.7) | 0.382 |  |
| Type of myeloma, no. (%) |  |  |  |  |
| IgG | 52 (58.4) | 56 (60.9) | 0.892 |  |
| Non-IgG | 37 (41.6) | 36 (39.1) |  |  |
| Type of CAR-T cell therapy, no. (%) | |  | 0.753 |  |
| BCMA | 27 (30.3) | 30 (32.6) |  |  |
| BCMA+ CD19 | 40 (44.9) | 28 (30.4) |  |  |
| Tandem BC19 | 22 (24.7) | 34 (37.0) |  |  |
| Previous therapy lines, median (range) | 29 (32.6) | 34 (36.9) | 0.527 |  |
| Previous HCT, no. (%) | 4 (1 - 10) | 4 (2 - 12) | 0.715 |  |

**Table S3.** Sensitivity analysis of key outcomes in the non-overlapping cohort (n = 92).

| Outcome |  | CONUT Subgroups (*P*-value) | PNI Subgroups (*P*-value) |
| --- | --- | --- | --- |
| Median PFS |  | Low vs. High: 18.2 vs. 12.9 months (0.012) | High vs. Low: 17.5 vs. 13.8 months (0.038) |
| Median OS |  | Low vs. High: NR vs. 45.6 months (0.021) | High vs. Low: NR vs. 43.9 months (0.019) |
| Incidence of PHT |  | 18.3% vs. 39.5% (0.008) | 16.7% vs. 35.2% (0.011) |
| Peak CAR transgene level |  | Higher in low CONUT (0.036) | Higher in high PNI (0.047) |

NR: Not reached; PHT: Prolonged hematologic toxicity.
